# Tuberculose de l'amygdale palatine simulant une lésion maligne et associant une miliaire tuberculeuse pulmonaire : à propos d'un cas à Ouagadougou, Burkina Faso

**DOI:** 10.48327/mtsi.v3i3.2023.422

**Published:** 2023-09-08

**Authors:** Moussa KADYOGO, Cheick Rachid BARGO, Franck Auguste H. A. IDO, Joséphine OUOBA, Boubacar MAMOUDOU, Christine N. MEDA, Aimé Sosthène OUÉDRAOGO, Assita SANOU LAMIEN, Moustapha SEREME

**Affiliations:** 1 Service d'ORL et de chirurgie cervico-faciale, Centre hospitalier universitaire de Bogodogo, Ouagadougou, Burkina Faso; 2 Service d'anatomie et de cytologie pathologique, Centre hospitalier universitaire de Tengandogo, Ouagadougou, Burkina Faso; 3 Service d'anatomie et de cytologie pathologique, Centre hospitalier universitaire Yalgado Ouédraogo, Ouagadougou, Burkina Faso; 4 Service d'ORL et de chirurgie cervico-faciale, Centre hospitalier universitaire pédiatrique Charles de Gaulle, Ouagadougou, Burkina Faso; 5 Service d'anatomie et de cytologie pathologique, Centre hospitalier universitaire de Bogodogo, Ouagadougou, Burkina Faso

**Keywords:** Tuberculose, Amygdale palatine, Anatomopathologie, Antibacillaire, Ouagadougou, Burkina Faso, Afrique subsaharienne, Tuberculosis, Palatine tonsil, Anatomopathology, Antibacillary therapy, Ouagadougou, Burkina Faso, Sub-Saharan Africa

## Abstract

La tuberculose amygdalienne correspond à la localisation infectieuse du bacille de Koch au niveau des amygdales palatines. Elle est rare. La tuberculose amygdalienne associée à une miliaire tuberculeuse est encore plus exceptionnelle.

**Objectif:**

Le but de notre travail est de rapporter un cas rare d'amygdalite tuberculeuse associée à une miliaire tuberculeuse pulmonaire.

**Patient et méthodes:**

Il s'est agi d'un cas de tuberculose amygdalienne associée à une miliaire tuberculeuse. Le motif de consultation principal était l'odynophagie chronique dans un contexte de consommation d'alcool et de tabac.

**Résultats:**

L'oropharyngoscopie mettait en évidence une amygdale droite hypertrophiée, ulcérée et hémorragique faisant penser à une lésion maligne. Une amygdalectomie à visée diagnostique avec examen anatomopathologique de la pièce opératoire a posé le diagnostic de tuberculose amygdalienne. La radiographie pulmonaire réalisée après obtention du résultat de l'examen anatomopathologique mettait en évidence la miliaire tuberculeuse. Le patient a bénéficié d'une polychimiothérapie antibacillaire pendant 6 mois. L’évolution était favorable et le patient a été déclaré guéri à l'issue du traitement. Il n'y a pas eu de récidive après 5 ans de recul.

**Conclusion:**

La tuberculose amygdalienne est rare. Celle associée à une miliaire pulmonaire est encore plus exceptionnelle. La biopsie amygdalienne pour examen anatomopathologique est suffisante pour le diagnostic. Il est nécessaire de demander une radiographie pulmonaire dans le bilan préopératoire avant toute biopsie amygdalienne ou toute amygdalectomie. L’évolution sous traitement antibacillaire est souvent favorable.

## Abstract (en)

Tonsillar tuberculosis is the infectious localization of Koch's bacillus in the palatine tonsils. It is rare. Tonsillar tuberculosis associated with miliary tuberculosis is even more exceptional.

**Objective:**

The aim of our work is to report a rare case of tuberculous tonsillitis associated with miliary tuberculosis.

**Patient and methods:**

This was a case of tonsillar tuberculosis associated with miliary tuberculosis. The main complaint was chronic odynophagia, which had been present for 7 months and was associated with weight loss. Questioning also revealed alcohol, tobacco and marijuana consumption.

**Results:**

Oropharyngoscopy revealed an enlarged, ulcerated and hemorrhagic right tonsil, suggesting a malignant lesion. Diagnostic tonsillectomy with anatomopathological examination of the surgical specimen led to the diagnosis of tonsillar tuberculosis. A postoperative chest X-ray revealed tuberculous miliaria. No other tuberculosis site was identified. No other confirmatory biological tests were carried out. The patient was treated with 4 anti-tuberculosis drugs (rifampicin, isoniazid, pyrazinamide, ethambutol) during 2 months and 2 anti-tuberculosis drugs (Rifampicin, Isoniazid) during 4 months. The evolution was favorable and the patient was declared cured at the end of treatment. There was no recurrence after 5 years.

**Conclusion:**

Tonsillar tuberculosis is rare. Tonsillar tuberculosis associated with pulmonary miliaria is even more exceptional. Tonsil biopsy for anatomopathological examination is sufficient for diagnosis. A chest X-ray should be requested as part of the preoperative workup prior to any tonsillar biopsy or tonsillectomy. GeneXpert (MTB/RIF) should be carried out if possible, not only for its value in the biological confirmation of tuberculosis but also to identify rifampicin resistance. Antibacillary treatment often leads to a favorable outcome.

## Introduction

La tuberculose amygdalienne correspond à la localisation infectieuse du bacille de Koch au niveau des amygdales palatines [7]. La tuberculose sévit à l’état endémique dans les pays pauvres. On assiste actuellement, dans les pays émergents, à une recrudescence de la tuberculose en général et des formes ORL en particulier à cause des difficultés socio-économiques et des déplacements de population [7]. Dans les pays développés, c'est plutôt l’épidémie de l'infection à VIH qui en est l'origine avec des formes résistantes, multi-récidivantes et polyviscérales.

Parmi les formes extra-pulmonaires, on distingue la tuberculose cervico-céphalique qui atteint principalement les ganglions cervicaux (87%), puis le larynx [8, 11]. La localisation au niveau des amygdales palatines est rare, même dans les pays d'endémie tuberculeuse [2, 9]. Elle est peu décrite dans la littérature.

**À** travers cette observation, nous rapportons un cas rare d'amygdalite tuberculeuse simulant une lésion maligne et dissimulant une miliaire tuberculeuse pulmonaire associée.

## OBSERVATION CLINIQUE

Monsieur K. I., âgé de 48 ans, né à Abidjan, est styliste de profession et réside à Ouagadougou. Il consulta dans le service d'ORL du CHU de Bogodogo en avril 2018 pour une dysphagie à type d'odynophagie qui semblait évoluer depuis 7 mois environ, associée à une otalgie unilatérale droite. Cette symptomatologie avait motivé plusieurs consultations dans des centres de santé de la place où plusieurs traitements avaient été prescrits sans résultat. Au contraire, l’état général du patient se dégradait avec un amaigrissement. Le patient n'a pas signalé de toux chronique. Monsieur K. I. est alcoolo-tabagique (10 paquets-année) et fume de la marijuana (3 à 4 fois par jour).

L'examen ORL au fauteuil avait permis de mettre en évidence un mauvais état buccodentaire avec une hypertrophie amygdalienne unilatérale droite, cryptique, ulcérée, nécrotique et saignant au moindre contact. Le sillon amygdalo-glosse homolatéral était également ulcéré. La région amygdalienne gauche était normale. L'examen cervical n'objectivait pas d'adénopathie cervicale.

Devant un tel tableau clinique, une lésion maligne amygdalienne droite a été suspectée. Nous avons décidé de réaliser une amygdalectomie bilatérale avec examen anatomopathologique de la pièce opératoire. L'amygdalectomie n'a pas été particulièrement hémorragique et les suites opératoires ont été simples. Le patient a cicatrisé normalement et a repris une alimentation normale 2 semaines après l'intervention.

**Figure 1:**
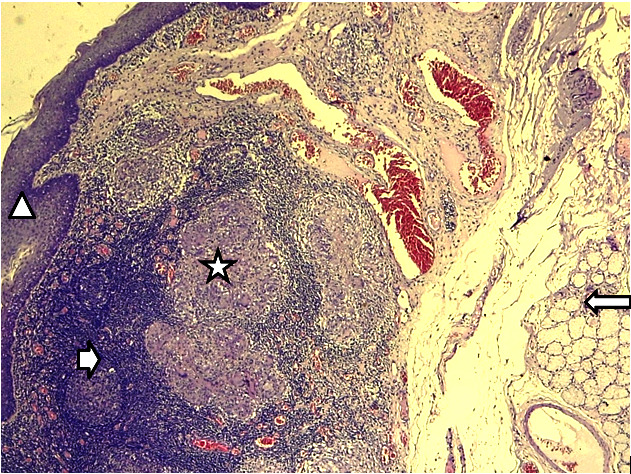
Gx50 coloration HES – Parenchyme amygdalien montrant un épithélium malpighien cryptique (triangle) surmontant des follicules lymphoïdes à centre germinatif clair (tête de flèche) et des glandes salivaires muqueuses (flèche). Ce tissu est le siège d'une réaction inflammatoire granulomateuse (étoile) Gx50 HES stain – Tonsil parenchyma showing cryptic squamous epithelium (triangle) overlying lymphoid follicles with clear germinal centers (arrowhead) and mucous salivary glands (arrow). This tissue is the site of a granulomatous inflammatory reaction (star)

**Figure 2:**
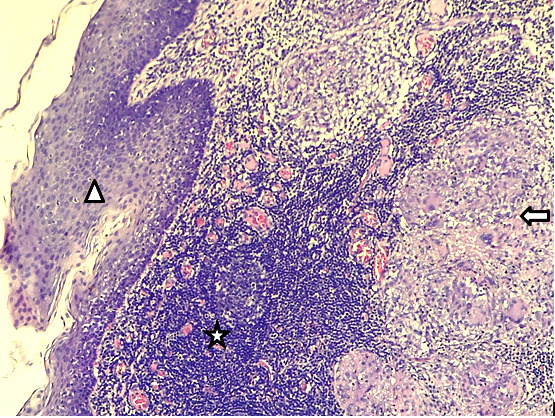
Gx100 coloration HE – épithélium malpighien cryptique (triangle) surmontant un tissu lymphoïde (étoile) siège d'une réaction inflammatoire granulomateuse folliculaire épithélio-giganto-cellulaire (flèche) Gx100 HE stain – cryptic squamous epithelium (triangle) overlying lymphoid tissue (star), the site of a follicular epitheliogiganto-cellular granulomatous inflammatory reaction (arrow)

**Figure 3:**
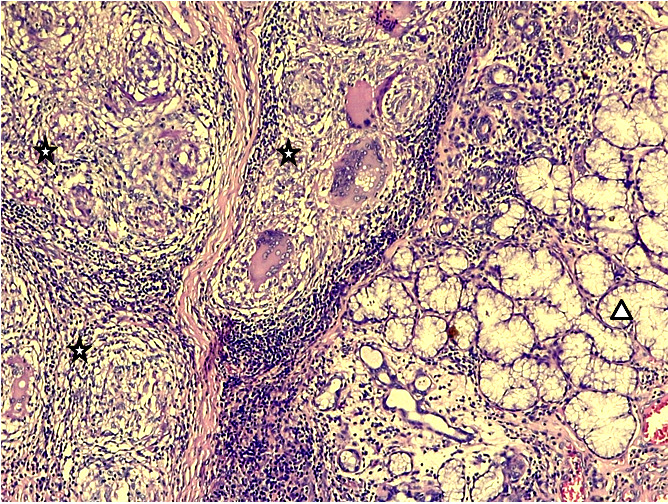
Gx200 coloration HE – inflammation granulomateuse folliculaire faite de cellules épithélioïdes et de cellules géantes de type Langhans limitées en périphérie par une couronne lymphocytaire (étoiles) à proximité de glandes salivaires muqueuses (triangle) Gx200 HE stain – follicular granulomatous inflammation made up of epithelioid cells and Langhans-type giant cells bordered on the periphery by a lymphocytic crown (stars) close to mucous salivary glands (triangle)

**Figure 4:**
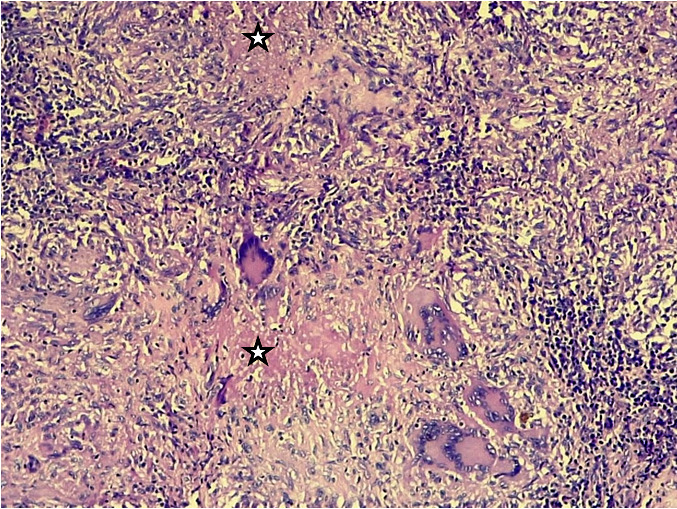
Gx100 coloration HE – follicules épithélio-gigantocellulaires avec couronne lymphocytaire centrant une nécrose éosinophile centrale incomplète (étoile) Gx100 HE stain – epithelio-giganto-cellular follicles with lymphocytic corona centering incomplete central eosinophilic necrosis (star)

L'examen histologique de la pièce opératoire a conclu à un aspect d'inflammation granulomateuse épithélio-giganto-cellulaire en faveur d'une tuberculose folliculaire (Fig. 1,2, 3,4). Il n'y avait pas de prolifération tumorale maligne sur le prélèvement.

**Figure 5:**
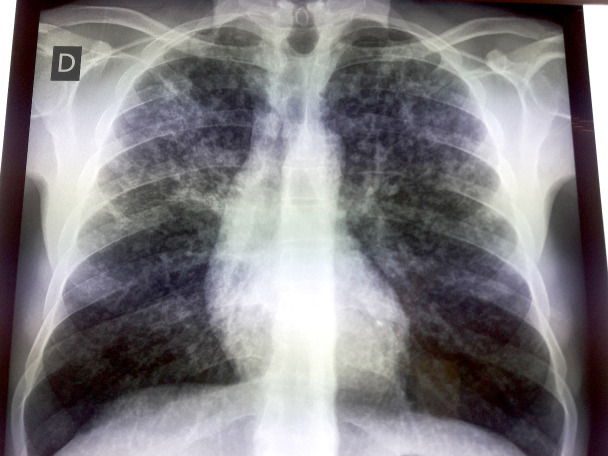
Radiographie des poumons de face, aspect de miliaire tuberculeuse Frontal chest X-ray, appearance of miliary tuberculosis

Nous avons alors décidé de compléter le bilan avec une radiographie des poumons de face. Elle a mis en évidence un aspect de miliaire tuberculeuse pulmonaire (Fig. 5). Il n'y a pas eu de recherche de bacille de Koch ni à l'examen direct ni à la culture ni par GeneXpert. Aucune autre localisation de tuberculose n'a été non plus retrouvée. La sérologie VIH était revenue négative.

Monsieur K. I. a été mis sous traitement antituberculeux selon le protocole en vigueur au Burkina Faso : 4 antituberculeux (rifampicine, isoniazide, pyrazinamide, **é**thambutol) pendant 2 mois et 2 antituberculeux (rifampicine, isoniazide) pendant 4 mois.

**Figure 6:**
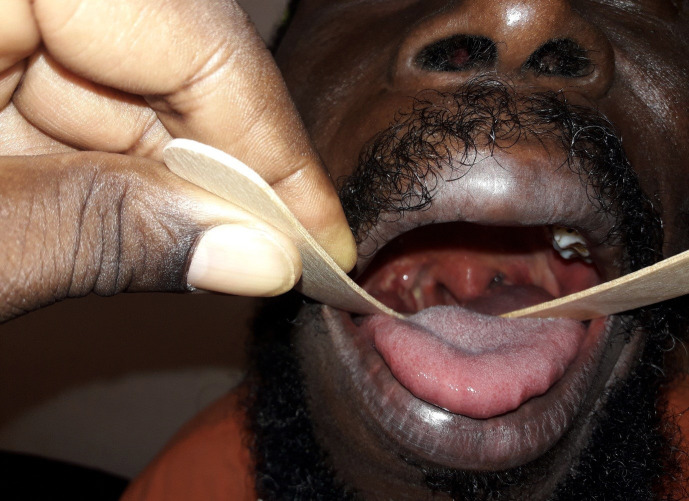
Oropharyngoscopie à l'abaisse-langue, contrôle de la région amygdalienne droite, évolution locale satisfaisante après 3 semaines Oropharyngoscopy with tongue depressor, control of the right tonsil area, satisfactory local evolution after 3 weeks

L’évolution était favorable avec cicatrisation de la région amygdalienne, et amélioration de la déglutition en 3 semaines (Fig. 6). Le patient était déclaré guéri à la fin du traitement. La radiographie de contrôle des poumons était revenue normale.

## Discussion

La tuberculose, une maladie infectieuse causée par le complexe *Mycobacterium tuberculosis*, peut être pulmonaire ou extrapulmonaire.

La tuberculose extrapulmonaire représente 25% de la morbidité tuberculeuse totale [1, 3, 10]. La tuberculose des amygdales est rare, son incidence est inférieure à 5%. Elle peut être primaire ou secondaire [6, 12].

La tuberculose amygdalienne est favorisée par les défauts de la muqueuse buccale due au tabagisme, par les prothèses dentaires traumatisantes ou par une mauvaise hygiène buccale, augmentant la virulence des mycobactéries [14, 15]. Dans notre cas le patient était alcoolo-tabagique (10 paquets-année) et fumait de la marijuana (3 à 4 fois par jour).

Le mal de gorge persistant, une déglutition douloureuse et un enrouement sont les symptômes locaux les plus courants de la tuberculose amygdalienne. À l'examen de l'oropharynx trois formes anatomocliniques peuvent être observées : la forme ulcéreuse chronique, la forme pseudonéoplasique comme observée dans notre travail et le « lymphome tuberculeux » (amygdale palatine hypertrophiée, pâle) [2, 11]. Les symptômes respiratoires sont généralement absents dans la tuberculose amygdalienne primaire, mais les symptômes généraux de la tuberculose, tels que la perte de poids, la fièvre persistante, les malaises, la cachexie et les sueurs nocturnes, ne sont observés que dans 37% des cas [5].

Les hémopathies malignes, telles que les lymphomes, ainsi que la sarcoïdose, la syphilis, l'angine de Vincent, la granulomatose de Wegner, doivent être prises en compte dans le diagnostic différentiel des maladies chroniques de l'amygdale [15].

La tuberculose de la tête et du cou représente près de 10% de tous les cas extrapulmonaires, les sites les plus courants étant les ganglions lymphatiques cervicaux suivis de la langue et du palais [13]. La tuberculose pulmonaire concomitante est observée chez environ 50% des patients atteints de tuberculose buccale [5]. Cependant, la tuberculose buccale n'est pas fréquente chez les patients atteints de tuberculose pulmonaire, bien que l'organisme infectieux passe par la cavité buccale chez les patients atteints de tuberculose pulmonaire [14]. Cela peut s'expliquer par le fait que la muqueuse buccale intacte agit comme une barrière naturelle et que les enzymes salivaires jouent également un rôle de mécanisme de défense [4]. Aussi plus de 50% des patients atteints d'une infection par le VIH et d'une tuberculose concomitante présentent une atteinte extrapulmonaire [5]. Chez notre patient la sérologie VIH était négative.

Le diagnostic de la tuberculose amygdalienne repose sur les résultats histopathologiques et l'identification des bacilles tuberculeux. Dans notre étude, nous avons pratiqué une amygdalectomie d'emblée parce que la tuberculose n’était pas fortement suspectée, nous avions plutôt **évoqué** une lésion maligne devant le terrain alcoolo-tabagique, l'aspect ulcéré, hypertrophié et hémorragique de l'amygdale palatine. Dans notre étude c'est donc l'anatomopathologiste qui a posé le diagnostic d'amygdalite tuberculeuse sur l'analyse de la pièce opératoire. La radiographie pulmonaire alors réalisée mettait en évidence la miliaire tuberculeuse. Le retard diagnostic chez notre patient peut s'expliquer par les multiples consultations dans des centres de santé primaires (Centre de santé et de promotion sociale, CSPS) où exerce un personnel soignant infirmier aux compétences limitées.

Dans notre contexte, en plus de l'examen anatomopathologique, la confirmation biologique de la tuberculose peut se faire à l'examen direct des prélèvements, ou à la culture. La réaction en chaîne de la polymérase (PCR) ou GeneXpert (MTB/RIF) a également une bonne sensibilité pour détecter les mycobactéries [5, 14]. La PCR en temps réel a l'avantage d'identifier la résistance à la rifampicine et est utile pour choisir le traitement le plus approprié. Pour notre cas, sur la base des résultats de l'examen anatomopathologique et de la radiographie des poumons, nous avons décidé d'instaurer un traitement antituberculeux.

Le traitement de la tuberculose amygdalienne est essentiellement médical, basé sur la polychimiothérapie antibacillaire sur une période de 6 mois. Dans notre étude, l'amygdalectomie a été à visée diagnostique et le patient a bénéficié par la suite d'un traitement antituberculeux conventionnel. L'amygdalectomie et le traitement médical ont permis alors de juguler la tuberculose amygdalienne et pulmonaire chez notre patient sans séquelle.

## Conclusion

La tuberculose amygdalienne associée à la miliaire tuberculeuse est exceptionnelle dans notre pratique. Il faut savoir y penser et demander une radiographie pulmonaire systématique dans le bilan préopératoire de toute amygdalectomie surtout chez l'adulte, de même qu'une analyse systématique histologique de toute pièce d'amygdalectomie. Le traitement est médical et repose sur les antituberculeux. L’évolution est le plus souvent favorable.

## CONTRIBUTION DES AUTEURS

Moussa KADYOGO : rédacteur principal du manuscrit, correspondant de l’étude Cheick Rachid BARGO, Boubacar MAMOUDOU, Joséphine OUOBA, Christine MEDA : ont participé à la rédaction de l'article Franck IDO, Aimé Sosthène OUÉDRAOGO, Assita SANOU LAMIEN : ont lu les lames et fourni l'iconographie en ce qui concerne l'anatomopathologie

Moustapha SEREME : a lu et corrigé le manuscrit

## LIENS D'INTÉRÊTS

Nous ne déclarons ni lien, ni conflit d'intérêts. Cette étude a été réalisée à nos propres frais. Nous n'avons reçu aucune contribution financière d'une tierce personne ou structure.
